# Communications

**Published:** 1873-06

**Authors:** 


*Communications.*

The following communications have been received: "Pneumonia Acute—Its Treatment," by A. S. Payne, M.D., of Virginia; "The Treatment of the Intestinal Disorders of Children," by S. Henry Dessan, M.D., Attending Physician to the New York Dispensary, etc., etc., of New York; "Gastric Hematamesis, Vicarious of Regular Menstruation Produced by Malarial Causes," by E. M. Vasser, M.D., of Alabama; "Medical Extracts—Practical and Philosophical, No. III.: Medical Philosophy," by L. B. Anderson, M.D., of Virginia; "Epileptic Mania Following the Suspension of Bromide Potash," by G. D. Hodge, M.D., of Arkansas; "Dislocation of the Spleen," by A. R. Kilpatrick, M.D., of Texas. Correspondence—by A. G. Smythe, M.D., of Mississippi; "Cases of Tetanus," by E. T. Henry, M.D., of Alabama.

Our friends are requested to forward their articles. All shall be attended to as promptly as possible. Will Dr. S. E. Winnemore, of Alabama, write out a fnll report of the interesting case communicated to us? Such a report will be better than any opinion we might suggest as to the nature of the case.

